# Effect of Freezing on Soybean Protein Solution

**DOI:** 10.3390/foods12142650

**Published:** 2023-07-09

**Authors:** Wenhui Li, Qiongling Chen, Xiaowen Wang, Zhenjia Chen

**Affiliations:** College of Food Science and Engineering, Shanxi Agricultural University, Jinzhong 030801, China; liwh0508@163.com (W.L.); cql_ttxs@163.com (Q.C.); wwxw11@163.com (X.W.)

**Keywords:** different freezing time, soybean isolate protein, 7S, 11S

## Abstract

To investigate the impact of frozen storage conditions on the physicochemical properties of soybean protein and explore the underlying mechanisms, this study focused on soybean isolate (SPI), ß-soybean companion globulin (7S), and soybean globulin (11S). The protein solutions were prepared at a concentration of 2% and subjected to freezing for 1 and 5 days. Subsequently, the protein content, physicochemical properties, secondary structure, sulfhydryl content, and chemical interaction forces were assessed and analyzed using UV spectrophotometry, Zeta potential measurements, SDS-PAGE, Fourier infrared spectroscopy, and endogenous fluorescence photoemission spectroscopy. The obtained results revealed that the solubility and total sulfhydryl content of SPI, 7S, and 11S exhibited a decreasing trend with prolonged freezing time. Among them, 11S demonstrated the largest decrease in solubility and total sulfhydryl content, followed by SPI, and 7S the least. During freezing, the aromatic amino acids of SPI, 7S, and 11S molecules were exposed, leading to increased hydrophobicity, protein aggregation, and particle size enlargement, and the structure of the protein changed from disordered structure to ordered structure. After freezing, the polarity of the microenvironment of SPI, 7S, and 11S increased, and their maximum fluorescence emission wavelengths were red-shifted. Notably, the largest red shift of SPI was from 332 nm to 335 nm. As freezing time increased, the contribution of hydrogen bonding increased, while the contribution of hydrophobic interactions decreased. This indicates that freezing affects the hydrophobic interactions, hydrogen bonding, and other chemical forces of the protein. The growth of ice crystals leads to the unfolding of protein molecular chains, exposure of internal hydrophobic groups, enhancement of hydrophobicity, and alters the secondary structure of the protein.

## 1. Introduction

**Figure 1:**
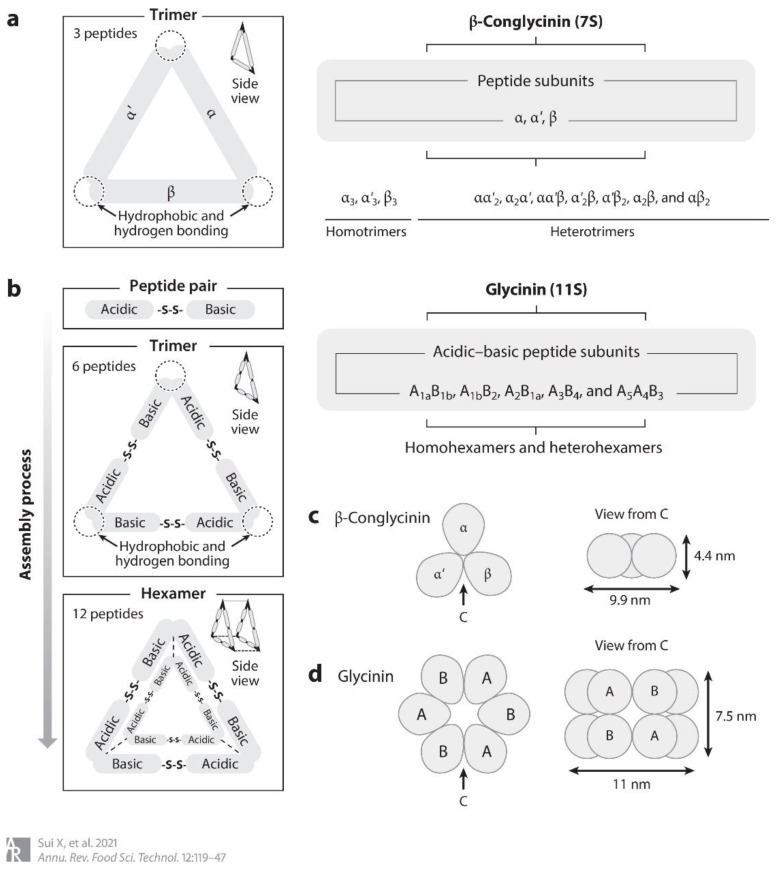
Molecular structure of 7S and 11S [8]. Note: (**a**) molecular structure characteristics of β-glycine; (**b**) molecular structure characteristics of glycine; (**c**) schematic diagram of 7S; (**d**) schematic diagram of 11S.

Soybean isolate protein is a highly nutritious food additive with a protein content exceeding 90%. It is typically extracted by alkali solubilization and acid precipitation methods [1]. According to the sedimentation coefficient, soy isolate protein can be divided into 2S, 7S, 11S, and 15S fractions [2]. The 7S globulin and 11S globulin comprise 27% and 34% of the soy proteins, respectively. The methionine and cysteine contained in soy protein isolate are three to four times that of other soy proteins [3]. The 7S protein, also known as ß-soybean globulin, is comprised of three subunits, α subunit (67 KDa), α′ subunit (71 KDa), and ß subunit (50 KDa) [4]. The sulphur-containing amino acid content of ß-soybean globulin is low, and the β subunit does not contain methionine, cysteine, and tryptophan residues. As a result, disulfide bonds are almost absent in the ß-soybean globulin molecule. The forces that stabilize its natural conformation are mainly hydrophobic interactions and hydrogen bonds [5]. On the other hand, 11S, also known as soy globulin, consists of two subunit groups, the acidic A subunit group (35–37 KDa) and the basic B subunit group (20 KDa). These subunits are connected by disulfide bridges and form a hexameric structure [6] (Figure 1). Due to their distinct functional properties and nutritional value, 7S and 11S are widely added to various products, such as infant formula and bread [7].

Soybean isolate protein has been viewed as a natural plant protein source [9], which has excellent solubility, emulsification, water retention, and gelling properties. It boasts a favorable composition of essential amino acids, promoting easy digestion and absorption. Incorporating soybean isolate protein into vegetarian and meat products not only enhances their nutritional value and functional properties but also helps reduce costs [10]. Adding soybean isolate protein to frozen pasta products can effectively reduce the cooking loss rate and enable a more uniform distribution of moisture in the food. The freezing process can inhibit the free migration of water in the food and prevent the formation of larger ice crystals. The addition of soybean isolate protein in frozen meat products can mitigate the occurrence of freezing cracks and improve the overall texture of the food. Moreover, research shows that in surimi products, soybean isolate protein cleaves the peptide chain of soy globulin by heating, exposing reactive groups. Assisted by disulfide bonds and hydrophobic forces, myofibrillar protein in fish, such as tilapia, binds more tightly to soybean isolate protein. This enhances gelation properties and increases water-holding capacity, improving the quality of the final product [11]. Given these advantages, soybean isolate protein is broadly used in the food industry [12]. Its utilization contributes significantly to the efficient utilization of protein resources and the enhancement of food quality.

Recently, freezing has become a widely used technique in food processing. It offers numerous benefits, including the preservation of fresh quality and taste, the inhibition of microbial growth, the suppression of enzyme activity, and the extension of shelf life. However, protein-rich foods are susceptible to damage during freezing due to the formation of ice crystals. This can impact both the macroscopic properties and microscopic structure of proteins, leading to changes in solubility and the exposure of internal reactive groups [13], thus changing their corresponding functional properties and causing the loss of nutrients.

Freezing and thawing processes cause irreversible damage to proteins, resulting in structural, conformational, or biological activity losses. Zhang et al. [14] investigated that after multiple freeze–thaw cycles, ice crystals reorganization and the freeze–thaw cycle accelerated protein oxidation, leading to alterations in protein secondary and tertiary structures and affecting internal water mobility. Zhao et al. [15] proposed that freeze–thaw cycles induce protein denaturation, increasing their surface hydrophobicity. And the freeze–thaw cycle can effectively improve the emulsification properties of soybean isolate proteins and affect the taste and quality of foods containing or supplemented with soybean isolate proteins. Fish proteins mainly exhibit a sharp decrease in solubility during freezing [16]. After freezing–thawing, the physical properties of the emulsion prepared by natural protein are mostly unstable, and even completely decompose into an oil and water phase [17]. Additionally, it has been reported that increasing freezing time will reduce the average particle size of soymilk, and that soluble soy protein solutions will become insoluble during the freezing process because of the formation of intermolecular disulfide bonds [18]. The water-holding capacity and soluble protein content of soybean isolate gels are reduced after freezing, and the hardness of the gel increases, which also affects the addition of soybean isolate in frozen products.

Protein molecules are substances with a spatial conformation under the combined action of covalent and non-covalent bonds. Protein denaturation primarily involves alterations in secondary and tertiary structures due to the disruption of non-covalent bonds [19]. In the context of freezing, protein denaturation is typically induced by alterations in solute concentration within the unfrozen aqueous phase during the formation of ice crystals [20]. When rapid freezing occurs, solute gradually concentrates in the unfrozen water phase, thus causing an increase in protein concentration in the surrounding unfrozen water, especially in slow freezing processes, where the protein is more susceptible to denaturation. Freezing dramatically alters the physical environment surrounding proteins, characterized by high crystallization concentrations, elevated salt ion levels, and an increased surface area of ice crystals. These changes result in protein unfolding and instability [21].

In this article, the purpose of the work was to investigate the impact of freezing for varying durations on the physicochemical properties and protein structures of soybean isolates, 7S, and 11S. To achieve this, we measured parameters such as particle size and Zeta potential distribution, soluble protein content, alterations in secondary and tertiary structures, and subunit distribution of the proteins. By analyzing these factors, we sought to obtain a comprehensive understanding of how freezing affects the properties and structural characteristics of soybean isolates, 7S, and 11S proteins.

## 2. Materials and Methods

### 2.1. Materials

The defatted soybean meal, with a protein content of 51%, was sourced from Jinyuan Grain and Oil Industry Co., Ltd. (Zhengzhou, China). Coomassie Brilliant Blue G250, Bovine Serum Albumin, β-Mercaptoethanol, SDS, Bromophenol Blue, Low Molecular Weight Protein Standard, Tris (hydroxymethyl) aminomethane, Tetramethylethylenediamine, dithiothreitol, and L-cysteine hydrochloride were purchased from Beijing Solebold Technology Co., Ltd. (Beijing, China). Sodium chloride, urea, sodium dihydrogen phosphate dihydrate, and disodium ethylenediaminetetraacetate dihydrate were obtained from Sinopharm Chemical Reagent Co., Ltd. (Shanghai, China). Sodium hydroxide and disodium hydrogen phosphate dodecahydrate were purchased from Tianjin Kaitong Chemical Reagent Co., Ltd. (Tianjin, China). Potassium sulfate was acquired from Tianjin Comio Chemical Reagent Development Center (Tianjin, China). Dithio-dinitrobenzoic acid was sourced from Aladdin Reagent Co., Ltd. (Shanghai, China). All other chemical reagents used in this study were of analytical grade.

### 2.2. Extraction of SPI

The defatted soybean meal was mixed with distilled water in a ratio of 1:15 (g/mL). The pH of the solution was adjusted to 9 using 1 N NaOH and stirred for 90 min. Subsequently, the homogenate was filtered through two layers of sterile gauze and centrifuged at 2800× *g* for 20 min. Then, the pH of the resulting supernatant was adjusted to 4.5 using 1 mol/L HCl to precipitate the proteins. After allowing it to stand for 30 min, the mixture was centrifuged at 400× *g* for 5 min, and the precipitate was collected and washed four times with water to make the salt fully dissolved. An appropriate amount of distilled water was added to the precipitate and stirred well, the pH was readjusted to 7, and the soybean protein isolate was obtained by vacuum freeze-drying [22].

### 2.3. Extraction of 7S and 11S

Based on the method of Nagano et al. [23], slight modifications were made. The defatted soybean meal was mixed with distilled water in a ratio of 1:15 (g/mL). The pH of solution was adjusted to 8.2 using 1 N NaOH and stirred for 90 min. Subsequently, the homogenate was filtered through two layers of sterile gauze and centrifuged at 2800× *g* for 20 min. Addition of sodium bisulfite to the above supernatant and stirring was continued for 10 min. And pH was adjusted to 5.4 using hydrochloric acid. The mixture was stirred for 20 min, left standing for 30 min, and centrifuged at 2800× *g* for 5 min. This process resulted in a mixture of 7S and 11S proteins.

The pH of supernatant was adjusted to 4.5 using 1 mol/L HCl and centrifuged at 1800× *g* for 5 min. The precipitate was collected and was washed three times with water. An appropriate amount of distilled water was added to the precipitate and stirred well, the pH was readjusted to 7, and 7S was obtained by vacuum freeze-drying.

The precipitate gained after centrifugation was washed with water and centrifuged at 2800× *g* for 5 min to ensure complete dissolution of salts. The appropriate amount of distilled water was added to the precipitate and stirred evenly. The pH value was readjusted to 7, and 11S was gained by vacuum freeze-drying.

### 2.4. Determination of Soluble Protein Content

The content of soluble protein was determined by the Coomassie brilliant blue method. Using bovine serum albumin as the standard sample, the absorbance values of bovine serum albumin solution at different concentrations were determined at 595 nm, and the standard curve of concentration and absorbance value was drawn. A 2% protein solution was prepared and stirred at room temperature for 2 h. Subsequently, the protein solution was frozen at −18 °C for 0, 1, and 5 days. After thawing the protein solution to room temperature, it was centrifuged at 10,000× *g* for 5 min, and the supernatant was collected at the same time. The soluble protein content was measured by spectrophotometer at a wavelength of 595 nm [24].

### 2.5. Viscosity

The viscosity of the protein solution was measured according to a modified method as reported by Qi, Xini et al. [25]. A 5% protein solution was prepared and stirred at room temperature for 2 h. Subsequently, the protein solution was frozen at −18 °C for 0, 1, and 5 days. After the protein solution was taken back to room temperature, 1 mL solution was taken for index determination. Using a CP50-1 plate mold (MCR102, Antona Co., Ltd., Graz, Austrian), the protein solution was placed on the rheometer induction plate, the plate spacing was 0.103 mm, the number of data points was 50, and the shear rate varied from 0.01 s^−1^ to 1000 s^−1^ following a logarithmic law. The viscosity of the protein solution was analyzed at different shear rates, providing valuable information on its flow properties.

### 2.6. Particle Size and Zeta Potential

A 2% protein solution was prepared and stirred at room temperature for 1 h. Then, the protein solution was centrifuged at 10,000× *g* for 10 min and filtered using a 0.45 µm filter membrane. The filtered solution was then stored in a refrigerator at −18 °C for 0, 1, and 5 days. The solution was taken out and balanced to room temperature and diluted with distilled water to a concentration of 1 mg/mL. The prepared samples were placed in a sample cell of the potential analyzer to set parameters. Water served as the dispersion medium, with refractive indexes of 1.450 for the protein and 1.333 for water. The average particle size, particle size distribution, and potential distribution of the soluble protein solution were measured after 120 s of equilibrium at 25 °C [26]. Particle size distribution and Zeta potential were measured with a Zetasizer Nano ZS90(Malvern Instruments Ltd., Malvern, UK) potentiometric analyzer. These measurements provide insights into the size and surface charge characteristics of the protein solution.

### 2.7. Sodium Dodecyl Sulfate-Polyacrylamide Gel Electrophoresis

A 2% protein solution was prepared and stirred at room temperature for 2 h. It was divided into separate portions and frozen at −18 °C for 0, 1, and 5 days. After thawing the protein solution back to room temperature, two different procedures were followed. For one portion of the protein solution, it was directly subjected to vacuum freeze-drying. Another portion of the protein solution was centrifuged at 10,000× *g* for 10 min, and then the supernatant and precipitate were gathered and subjected to vacuum freeze-drying.

The samples obtained by vacuum freeze-drying were added to the prepared electrophoretic sample at a concentration of 1 mg/mL. The electrophoresis method outlined by Laemmli et al. [27] was slightly modified. Separation gels and concentrated gels were prepared at 12% and 5% concentrations, respectively. The separation gel was subjected to a voltage of 150 V, while the concentrated gel was subjected to a voltage of 100 V during electrophoresis. After electrophoresis, the gel was immersed in a fixation solution and left overnight. Then, the gels were stained with Coomassie Brilliant Blue G-250 for 3 h. After staining, the gels were destained with distilled water and then scanned with a CanoScan scanner (Cano Scan LiDE 300, Canon Corporation, Japan). In reduction electrophoresis, 2% ß-mercaptoethanol was added to the sample buffer, and the subsequent steps were the same as in non-reducing electrophoresis. This procedure allowed for the visualization and analysis of protein bands, providing insights into the composition and molecular weight of the proteins under different freezing conditions.

### 2.8. UV Spectrum

A 2% protein solution was prepared and stirred at room temperature for 1 h. Afterward, the protein solution was centrifuged at 10,000× *g* for 5 min and filtered using a 0.45 µm filter membrane. The filtered solution was deposited in a refrigerator at −18 °C for 0, 1, and 5 days. Subsequently, the solution was taken out and balanced to room temperature. To analyze the protein content, the protein solution was diluted and scanned by Agilent Cary60 ultraviolet-visible spectrophotometer (Agilent Technology Co., Ltd., Beijing, China). Samples were taken from 200 nm to 400 nm for scanning, acquiring spectra at a resolution of 0.2 nm with a scan rate of 100 nm/min [28]. This spectrophotometric assay provided valuable information about the absorbance profile of the protein solution at different wavelengths, allowing for the evaluation of protein concentration and potential changes in its structure or composition due to the freezing process.

### 2.9. Intrinsic Fluorescence

A protein solution with a mass fraction of 2% was prepared, then stirred for 1 h at room temperature. Subsequently, the solution was stored in a refrigerator at −18 °C for 0, 1, and 5 days. After removing the solution from the refrigerator, it was allowed to reach room temperature and then diluted with distilled water to a concentration of 0.1 mg/mL, based on the method of Wang et al. with slight modifications [29]. To analyze the fluorescence characteristics of the protein solution, an excitation wavelength of 280 nm was set and the fluorescence emission spectrum was scanned from 300 to 400 nm. The slit width was set at 5 nm, and the scanning rate was maintained at 200 nm/min. This fluorescence spectroscopy analysis provides insights into the intrinsic properties of the proteins, such as their structural conformation and interactions, by monitoring their fluorescence emission at different wavelengths. By examining the changes in the emission spectrum, potential alterations in the protein structure and environment due to the freezing process can be evaluated.

### 2.10. Fourier Transform Infrared (FTIR)

A 2% protein solution was prepared and stirred at room temperature for 2 h. Subsequently, the solution was frozen at −18 °C for 0, 1, and 5 days. After thawing the protein solution to room temperature, it was divided into two parts for further analysis. One part of the protein solution was directly subjected to vacuum freeze-drying, while the other part was centrifuged at 10,000× *g* for 10 min. After centrifugation, the supernatant and precipitate were separately collected and subjected to vacuum freeze-drying. 

Following the method of Shin et al. [30], to determine the protein secondary structure, the dried samples were precisely weighed (2 mg) and mixed with 200 mg of KBr. The mixture was finely ground into a powder and pressed into transparent slices. Infrared spectrometry (Tensor 27, Bruker, Germany) was then performed on the sample, scanning the infrared spectrum in the range of 4000–400 cm^−1^ with a resolution of 4 cm^−1^ and 32 scanning cycles. Each group of samples was measured three times to ensure accuracy. The spectral data obtained from the amide I band (1700–1600 cm^−1^) were analyzed using Peak Fit 4.12 software (PeakFit v4.12, SeaSolve Software Inc., USA). This analysis provides insights into the protein’s secondary structure, allowing for the identification and quantification of structural components such as α-helix, β-sheet, and random coil. By comparing the infrared spectra of the protein samples subjected to different freezing durations and processing methods, any changes in the secondary structure can be evaluated, providing valuable information about the impact of freezing on protein conformation and stability.

### 2.11. Total Sulfhydryl Group Contents

Modified slightly according to the method of Ruan et al. [31], SPI, 7S, and 11S proteins were added to reaction buffer B, which consisted of a 0.1 mol/L pH 8.0 sodium phosphate buffer containing 1 mmol/L EDTA and 8 M urea. The protein concentration was adjusted to 2% and the solution was stirred at room temperature for 2 h. The stirred protein solution was frozen in a refrigerator at −18 °C for 0, 1, and 5 days. The protein solution was taken out and balanced to room temperature and was centrifuged at 10,000× *g* for 10 min. The supernatant (5.5 mL) was collected and was mixed with 0.1 mL Ellman’s reagent solution. The mixture was then stored at room temperature for 15 min in the dark. To measure the absorbance, the samples were subjected to ultraviolet spectrophotometry (Shanghai Jinghua Technology Co., Ltd., Shanghai, China) at a wavelength of 412 nm. This measurement allowed for the quantification of the reaction product formed between Ellman’s reagent and the released sulfhydryl groups, providing insights into the protein’s thiol content. By comparing the absorbance values obtained from the protein samples subjected to different freezing durations, the impact of freezing on protein stability and thiol content can be assessed. Higher absorbance values indicate a higher concentration of thiol groups, reflecting the integrity and reactivity of the proteins.

### 2.12. Determination of Protein Action Force

Based on the methodology described by Gómez-Guillén et al. [32], with some improvements in this research, SPI, 7S, and 11S proteins were dissolved in different solutions: 0.05 mol/L NaCl (SA), 0.6 mol/L NaCl (SB), 0.6 mol/L NaCl + 1.5 mol/L urea (SC), 0.6 mol/L NaCl + 8 mol/L urea (SD), and 0.6 mol/L NaCl + 8 mol/L urea + 0.05 mol/L dithiothreitol (SE). The protein concentration was set at 2% and stirred at room temperature for 2 h. Subsequently, the protein solutions were placed in a refrigerator at −18 °C for 0, 1, and 5 days. The balance was taken out to room temperature and centrifuged at 10,000× *g* for 10 min. The soluble protein content in the supernatant was determined by Coomassie brilliant blue method. The contributions of specific bonding types were evaluated as follows: the ionic bond contribution was determined by the difference of protein content between SB and SA solutions, the hydrogen bond contribution was determined by the difference between SC and SB solutions, the hydrophobic interaction contribution was determined by the difference between SD and SC solutions, and the disulfide bond contribution was determined by the difference between SE and SD solutions. This analysis allowed for the quantification of the individual bonding contributions in the solubility of the proteins, offering insights into the role of ionic, hydrogen, hydrophobic, and disulfide bonds in the protein stability under different solution conditions.

### 2.13. Statistical Analyses

Data processing was performed using Microsoft Excel 2019 (Microsoft Corp, Redmond, Washington, DC, USA), while drawing of graphs and plots was conducted using Origin 2018 (Origin Lab, CA, USA). For the statistical analysis of the data, SPSS 18.0 software (SPSS Inc., Chicago, IL, USA) was employed. Variance analysis was conducted using one-way ANOVA, and the Duncan method was applied for post hoc multiple comparisons. The confidence interval used for the analysis was set at 95%, ensuring a reliable assessment of the statistical significance of the results.

## 3. Results and Discussion

### 3.1. Solubility

During freezing, soy protein isolate (SPI) is highly susceptible to protein denaturation and aggregation, leading to a decrease in protein solubility. Protein solubility serves as a crucial indicator of freeze-induced protein denaturation. Wang et al. [33] observed that the solubility of protein in grass carp decreased with the prolongation of storage time when different cryoprotectants were added. Similarly, Hashizume [34] found that the solubility of SPI solution decreased to 50% after 10 days of freezing at −5 °C, and the precipitated substance could be dissolved by mercaptoethanol, thus indicating that SPI formed insoluble aggregates through disulfide bonds during freezing. The decrease in protein solubility during frozen storage was attributed to the formation of hydrophobic interactions, ion interactions, hydrogen bonds, and disulfide bonds.

**Figure 2:**
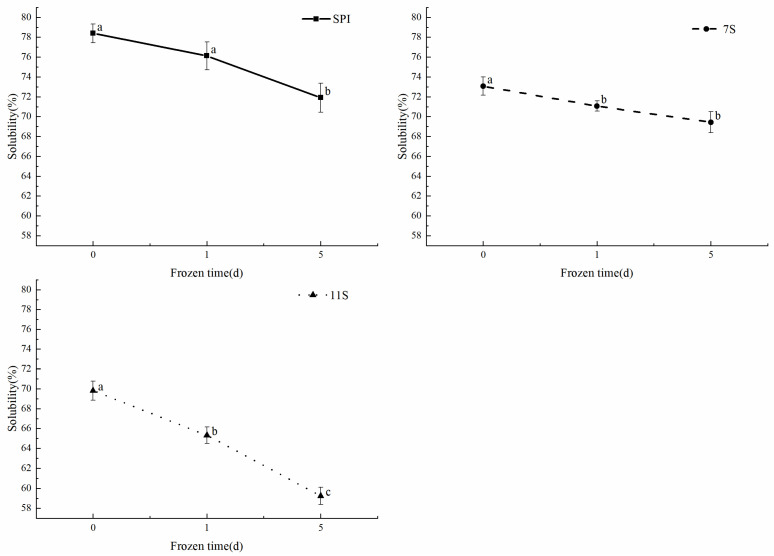
Distribution of soluble protein content in different protein frozen storage days. Note: F0 represents 0 days of freezing, F1 represents 1 day of freezing, and F5 represents 5 days of freezing. Different lowercase letters on the shoulders of the same indicator indicate significant differences (*p* < 0.05).

Figure 2 illustrates the decrease in protein solubility after freezing. Unfrozen SPI exhibited the highest solubility, reaching 78.38%. In contrast, 11S had the lowest solubility at 69.82%, and its solubility decreased significantly after 5 days of freezing. Freezing has the greatest effect on the solubility of 11S, likely due to the protein denaturation and changes in chemical forces within the protein molecules, causing the formation of insoluble proteins and decreased solubility. After freezing, the solubility of SPI, 7S, and 11S solutions all decreased to different degrees, with 11S showing the largest decrease, followed by SPI, with 7S showing the smallest change. Since SPI primarily consists of 7S and 11S, the freezing characteristics of SPI solutions are directly influenced by the freezing properties of the 7S and 11S solutions. Figure 2 clearly shows that it can be seen that the 11S solution has the greatest variation in freezing properties, which is the main reason for the variation in the freezing properties of the SPI solution. The changes in the 11S solution during freezing are closely related to the structural properties of its protein molecules, which have cold-sinking properties [5]. In contrast, the solubility of 7S solution changed slowly before and after freezing, which possibly related to the binding of protein molecules to some sugar chains, and its sugar content was 3.8–5.4% [35]. Although SPI is not a simple mixture of 7S and 11S, but rather composed in a specific way through valence bonding, it is evident that the solution properties of 7S and 11S also significantly affect SPI, exemplifying the variations in freezing characteristics.

### 3.2. Viscosity

The viscosity of protein solutions is primarily determined by the interactions between proteins and water, as well as protein–protein interactions [36]. The viscosity of soy protein solutions is affected by the degree of hydration between proteins and water, and it is directly proportional to the extent of hydration. Generally, the hydration capacity of protein will increase after denaturation, but if denaturation leads to protein aggregation, the hydration capacity of protein will decrease due to the strengthening of protein–protein interaction [37]. The decrease in viscosity with increasing shear rate is a representative characteristic of pseudoplastic or shear-thinning fluids [38]. 

**Figure 3:**
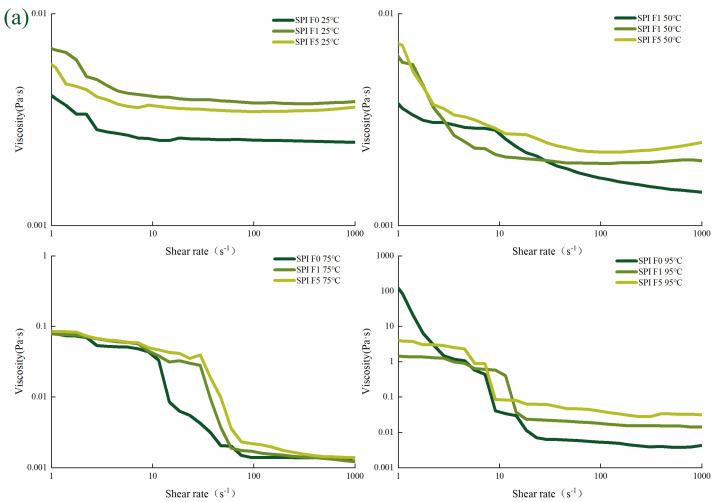

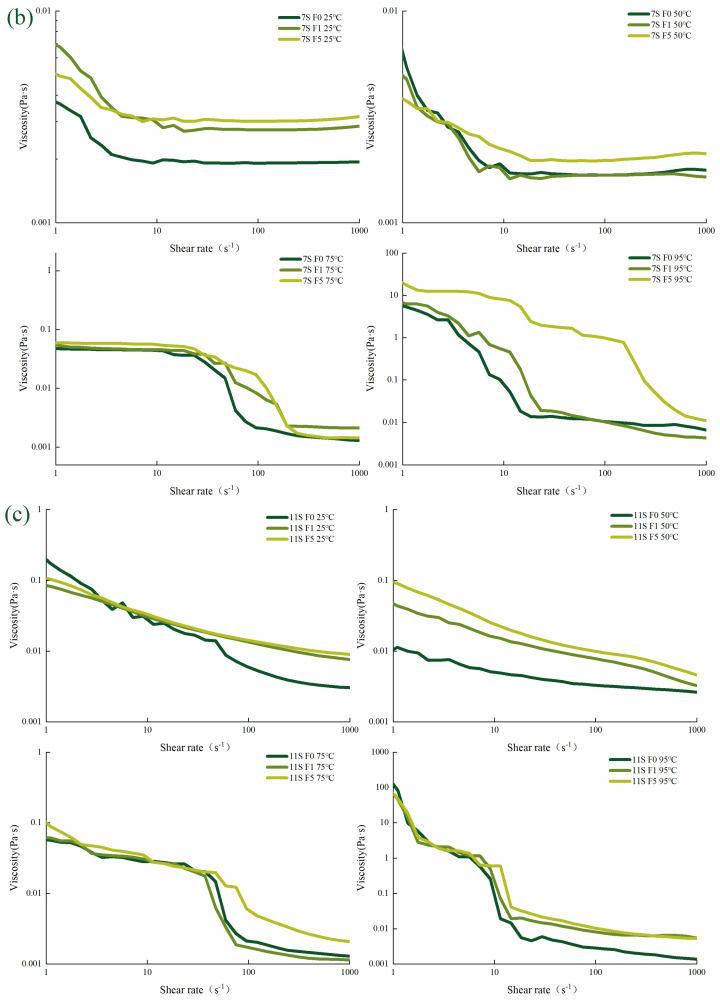
Relationship between viscosity and shear rate of different proteins at different tempera-tures. Note: (**a**) the relationship between viscosity and shear rate of SPI at different temperatures; (**b**) the relationship between viscosity and shear rate of 7S at different temperatures; (**c**) the relationship between viscosity and shear rate of 11S at different temperatures.

As depicted in Figure 3, the viscosity of 11S solution was the highest, followed by SPI and 7S solution. The viscosity of protein solution decreased with higher shear rates. Under the influence of high shear force, asymmetrically dispersed molecules have a tendency to align themselves along the direction of the shear surface [39]. This alignment disrupts the intermolecular connections within the protein chains, leading to a weakened protein structure and reduced frictional resistance. Consequently, the solution flows more smoothly and exhibits lower viscosity [40]. Furthermore, it was observed that the viscosity of the protein solution gradually increased with prolonged freezing. The freezing process compromised the natural stability of the protein, possibly due to substance rearrangement [41]. During freezing, the protein–water interactions were strengthened by hydrogen bonding, while the formation of ice crystals disrupts these interactions. This, in turn, enhances the protein–protein interactions and contributes to the increased viscosity of the protein solution. Simultaneously, the viscosity of the protein solution tended to increase as the temperature increased. This phenomenon can be attributed to the denaturation of the protein’s internal structure at high temperatures, resulting in the unfolding of molecular chains and enhanced protein–protein interactions, ultimately leading to an increase in viscosity. The impact of temperature on the viscosity of pea protein has also been examined, revealing that temperatures exceeding 75 °C induce denaturation of the native protein, leading to a decrease in protein solubility and consequently an increase in viscosity [42].

### 3.3. Particle Size and Zeta Potential Analyses

Particle size is a crucial parameter for characterizing the size of soluble proteins, and it can indicate the degree of protein aggregation under different conditions. Typically, an increase in protein particle size confirms an upward trend in protein aggregation.

**Figure 4:**
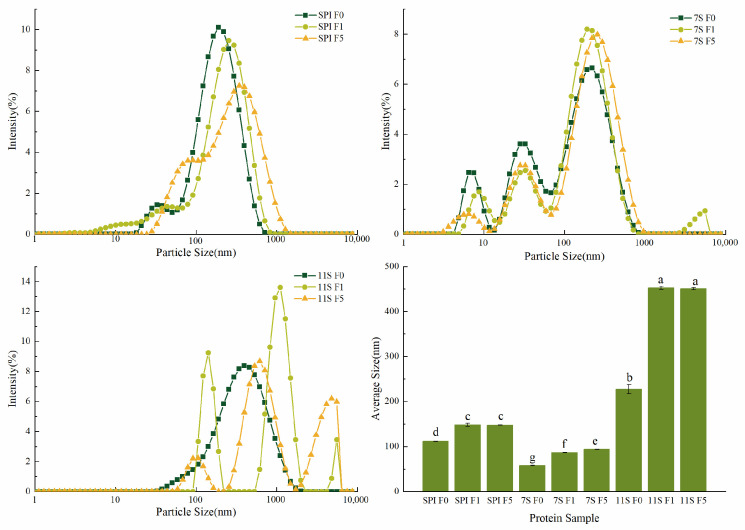
Particle size distribution and average particle size distribution of different protein solutions frozen for different days. Note: different lowercase letters on the shoulders of the same indicator indicate significant differences (*p* < 0.05).

As illustrated in Figure 4, the overall curve of SPI particle size shifted to the right with increasing freezing days. The average particle size increased significantly, and the particle size range was mainly concentrated between 100–1000 nm. Yu, Jie et al. demonstrated that following two freeze–thaw cycles, the particle size of soy protein isolate emulsion increased, indicating poor freeze–thaw stability [43]. The particle size distribution of 7S included three peaks. After freezing, the peak value of the short peak with smaller particle size on the left side decreased, and the peak value of the large peak with larger particle size on the right side increased, and the particle size also increased significantly. Notably, when 11S was not frozen, its particle size distribution exhibited a more concentrated range, primarily between 100 nm and 1000 nm. Moreover, the average particle size of 11S was the largest, followed by SPI, with 7S being the smallest. This can be attributed to the higher content of hydrophobic amino acids present in 11S itself. These amino acids enhance the interaction between protein and protein, resulting in protein aggregation and weakened protein–water interactions [44], consequently resulting in low protein solubility and large particle size. For 11S, after freezing, the particle size distribution transformed from a single continuous peak to three separate peaks, and the distribution range shifted to the right as a whole. At 5 days of freezing, the particle size distribution was primarily concentrated beyond 1000 nm, accompanied by a significant increase in particle size. Notably, the average particle size of 11S was the largest, because 11S is a hexamer and it has the characteristic of ‘cold sink ’. Consequently, 11S is more likely to produce large aggregates during freezing, which has a more significant impact on soy protein isolate during freezing. During freezing of soy protein, the protein structure unfolds, leading to an increase in the protein’s surface area. This, in turn, strengthens hydrogen bonds and hydrophobic interactions among the proteins, leading to protein aggregation and an overall increase in particle size [45].

It is essential to note that the protein solution used for particle size determination should be soluble, reflecting the particle size distribution in soluble proteins. Although the increase in particle size only indicates the extent of soluble protein aggregation, it still provides valuable insights into the trend of protein aggregation under freezing conditions. Therefore, the particle size distributions of the three proteins before and after freezing are in agreement with the solubility changes in Figure 2.

Zeta potential is related to the charge strength of the ion surface in a colloidal particle, providing insights into the electrostatic repulsion or gravitational forces within a colloidal dispersion system. Generally, the more positive or negative charges on the molecular surface, the greater the intermolecular repulsion and the smaller the tendency for aggregation and sinking to occur between colloidal particles.

**Figure 5:**
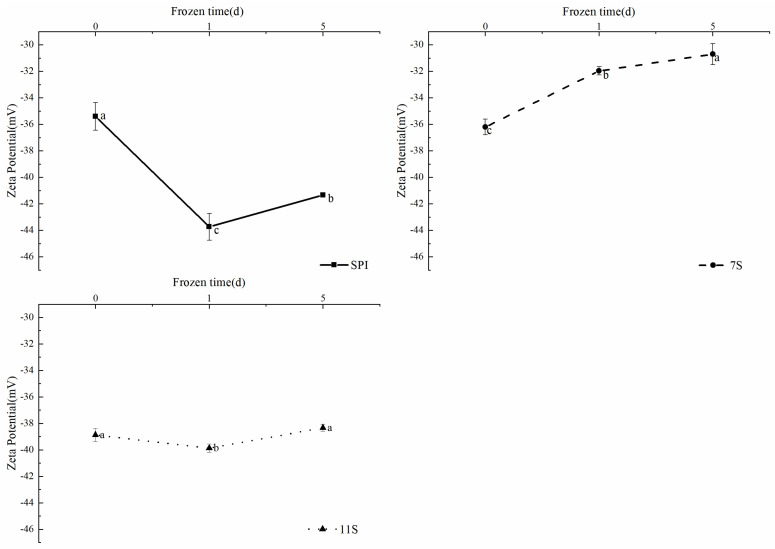
Potential distribution of different protein solutions frozen for different days. Note: different lowercase letters on the shoulders of the same indicator indicate significant differences (*p* < 0.05).

Research by Song et al. [46] demonstrated that the larger the absolute value of the Zeta potential, the more favorable the solubilization, dispersion, and stabilization of the proteins. As depicted in Figure 5, the Zeta potentials of the three proteins exhibited negative values before and after freezing. Among them, SPI had the smallest absolute potential value, while 11S had the largest absolute potential value in its unfrozen state. Following freezing, the absolute value of SPI potential increased, that of 7S potential decreased, and the absolute value of 11S potential remained relatively unchanged. Freezing induced varying degrees of protein denaturation. The soluble parts of SPI and 11S aggregated, and the structure was more stable, which reduced the overall trend of further aggregation. Conversely, 7S exhibited the opposite behavior.

However, freezing-induced protein aggregation, leading to an increase in particle size, resulted in the formation of soluble aggregates that counteracted the propensity for further protein aggregation and precipitation. This effect is manifested as an elevation in Zeta potential values.

### 3.4. UV Spectra and Intrinsic Fluorescence Emission Spectra

UV absorption spectra is utilized to characterize changes in aromatic amino acid residues (such as tryptophan Trp, tyrosine Tyr, and phenylalanine Phe) within proteins. These spectra can reveal the denaturation or unfolding of proteins and the exposure of previously hidden amino acids [47]. Based on the absorption of UV spectra, the conformational changes of protein molecules can be inferred [48].

**Figure 6:**
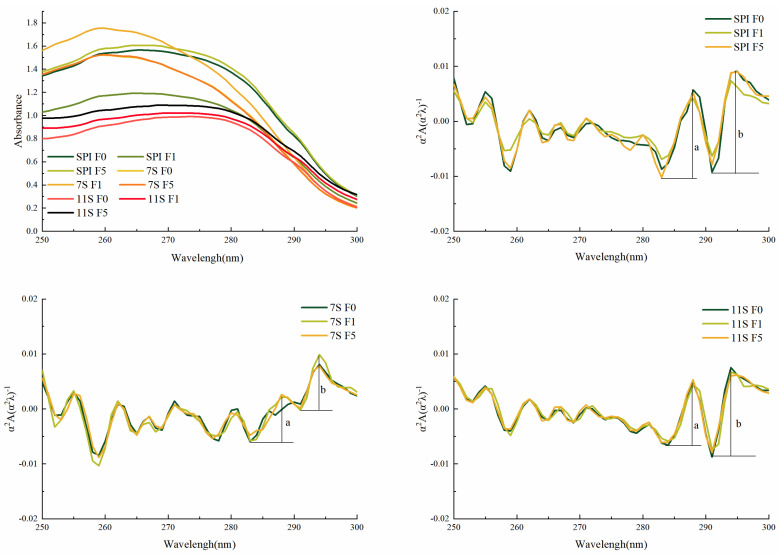
UV absorption spectra and second-derivative spectra of different protein solutions frozen for different days. Note: a and b represent the difference between the two wave peaks and troughs.

The UV absorption spectra and second-derivative spectra of various protein solutions frozen for different days are shown in Figure 6. In the unfrozen state, the valley peak of SPI is 264 nm, the valley peak of 7S is 260 nm, and the valley peak of 11S is mainly concentrated at 274 nm. This suggests that 11S exhibits higher polarity and hydrophobicity compared to SPI and 7S. In the second-derivative spectra, the protein has two positive absorption and two negative absorption peaks in the range of 280–300 nm. The positive absorption peaks are observed at 287 nm and 293 nm, while the negative absorption peaks are 282 nm and 290 nm [49]. The peak at 287 nm is attributed to the combined effect of tyrosine and tryptophan, whereas the peak at 296 nm is solely attributed to tryptophan [50].

The alterations in protein’s three-dimensional structure and the exposure of tyrosine residues can be reflected by calculating the ratio r = a/b. The smaller the r value, the lower the polarity of the microenvironment in which the protein is located, and the hydrophobic groups are exposed. As freezing time increases, the r values for SPI were 0.874, 0.810, and 0.786, for 7S they were 0.796, 0.782, and 0.751, and for 11S they were 0.752, 0.733, and 0.712, respectively. After 5 days of freezing, with the decrease of r value, the polarity of the microenvironment of protein tyrosine decreased, the hydrophobic groups were exposed, and hydrophobicity was enhanced. This change is attributed to the freezing of water during the freezing process, which causes the exposure of some hydrophobic groups and an increase in hydrophobicity.

Fluorescence spectroscopy is primarily employed to detect tertiary structural changes of proteins [51] and is commonly used for analyzing the exposure index of aromatic amino acids. The endogenous fluorescence of tryptophan residues is very sensitive to the polarity of the protein’s microenvironment, and protein aggregation leads to a decrease in the distance between groups within the protein molecule, thus causing a change in the nature of the endogenous fluorescence. 

**Figure 7:**
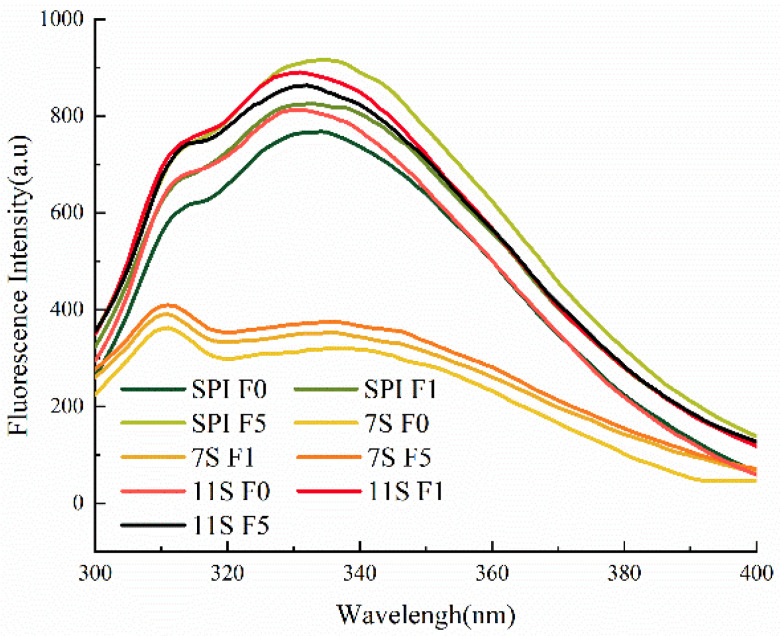
Fluorescence emission spectra of protein solution frozen for different days.

When unfrozen, the maximum fluorescence emission wavelength of SPI and 7S was 332 nm, while for 11S it was 330 nm. From Figure 7, it can be observed that the fluorescence emission wavelength of SPI changed to 334 nm after 1 day of freezing, and to 335 nm after 5 days of freezing. Similarly, 7S and 11S were also red-shifted and the fluorescence intensity increased. These findings indicate that the degree of exposure of protein molecules in solution increases during freezing. When the red shift in 7S fluorescence is substantial, it suggests that more free sulfhydryl groups become exposed in the spatial structure, leading to the formation of additional disulfide bonds. Moreover, tryptophan residues initially buried in a hydrophobic environment become exposed to the hydrophobic environment, resulting in an increase in the polarity of the protein’s microenvironment. In a relatively hydrophobic environment, the maximum fluorescence emission wavelength of tryptophan is usually located between 331–347 nm. The results presented in Figure 7 indicate that the tryptophan residues, located in a hydrophobic environment, experienced a red shift in their maximum fluorescence emission wavelengths after freezing. This red shift signifies the exposure of hydrophobic groups, which could contribute to the formation of protein aggregates through hydrophobic interactions [14]. Consequently, a decrease in protein solubility and an increase in particle size occur. Feng et al. [52] also reported a corresponding red shift in the maximum fluorescence emission wavelength of peanut proteins after multiple freeze–thaw cycles, and the tryptophan residues were partially exposed to polar environments.

### 3.5. SDS–Polyacrylamide Gel Electrophoresis

**Figure 8:**
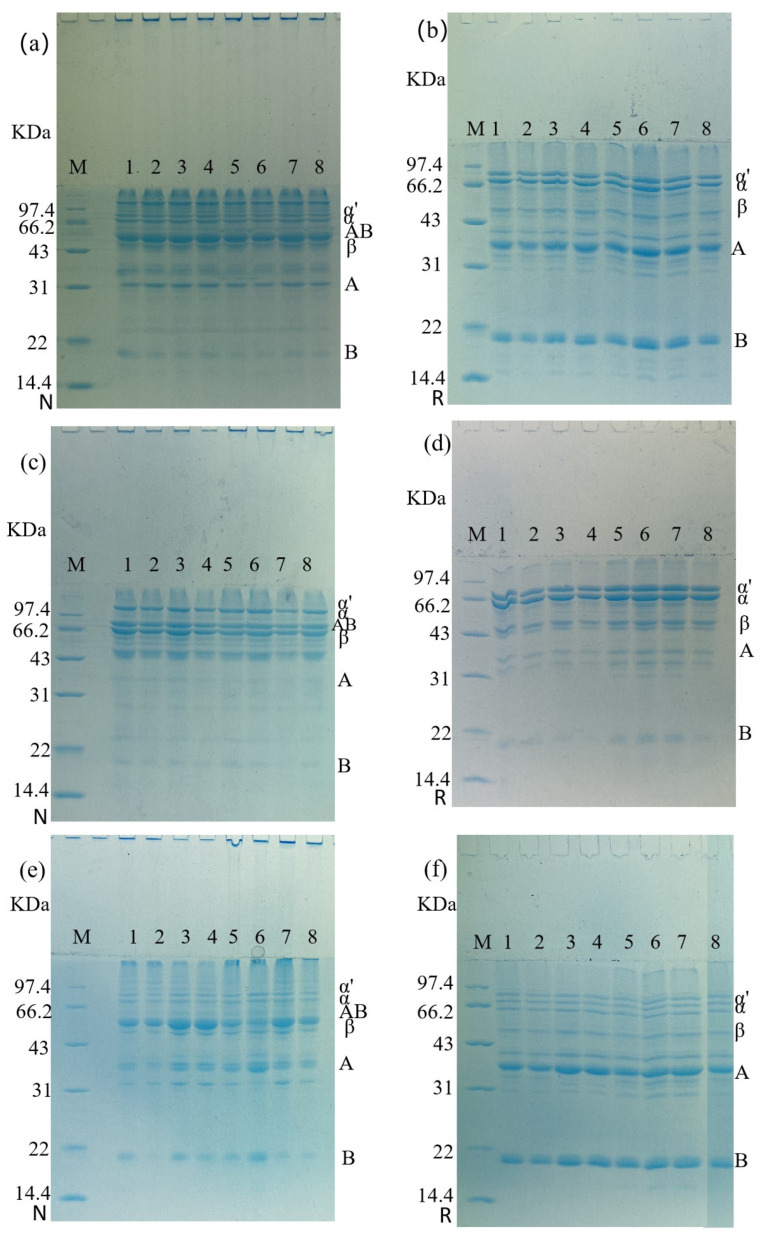
Electrophoretic profiles of non-reduced (N) and reduced (R) protein solutions at different days of freezing. Note: (**a**) SPI non-reduction profile; (**b**) SPI reduction profile; (**c**) 7S non-reduction profile; (**d**) 7S reduction profile; (**e**) 11S non-reduction profile; (**f**) 11S reduction profile. Lane M: protein specimen. Lanes 1–8: protein powder, supernatant F0, supernatant F1, supernatant F5, precipitate F0, precipitate F1, precipitate F5, protein powder.

As depicted in Figure 8a,b, there were no significant changes in the subunit bands of the SPI supernatant and precipitate after freezing when they were not reduced. Additionally, the B subunit band of the precipitated portion was lighter than the supernatant band. The conspicuous coloration above the electrophoresis bands indicates the presence of large protein aggregates in the solution. After reduction, the electrophoresis bands of A and B subunits deepened, indicating that AB subunit was connected by disulfide bond. In Figure 8d, the electrophoretic profile of the supernatant solution displayed a lightening of the A subunit band and a deepening of α. Conversely, the precipitated part of the subunit bands was darker, and the B subunit bands gradually deepened with the increase of freezing time. The remaining bands did not change significantly. Moving to Figure 8e, with the increase of freezing time, the AB subunit bands became thicker and B subunit bands became less pronounced. Furthermore, in Figure 8f, the A subunit electrophoretic bands gradually widened with increasing freezing time, indicating that 11S was more prone to aggregation and precipitation.

During the extraction of 7S, a reducing agent was added to break the disulfide bond to separate 7S and 11S. However, some remnants of 11S may still be present. In the non-reduction electrophoresis map of Figure 8, the distribution of the supernatant and precipitate bands was similar. However, in the reducing electrophoresis map, the supernatant electrophoresis pattern contained only a small amount of 11S A subunit, whereas the precipitate contained more 11S A subunit and B subunit. This indicates that the remaining 11S subunit in 7S during freezing is more likely to aggregate and enter the precipitation, which laterally demonstrates the difference in freezing characteristics of the two proteins during freezing.

### 3.6. Secondary Structure Analysis

Fourier transform infrared (FTIR) spectroscopy is a classical method for protein conformation analysis [53]. In freezing conditions, freezing denaturation can lead to alterations in the secondary structure of the proteins. Different peaks in the FTIR spectrogram represent different groups. For instance, the range of 3250–3400 cm^−1^ is associated with hydroxyl groups, while the aliphatic amino acid residues exhibit stretching modes around 2800–3000 cm^−1^ and bending modes around 1440–1465 cm^−1^ for C-H bonds. The amide bond in proteins has different vibration modes, with the amide I band and the amide III band being crucial for assessing the secondary structure [54]. The amide I band consists of bands in the range of 1600–1700 cm^−1^, 1608–1622 cm^−1^, 1622–1637 cm^−1^, 1637–1645 cm^−1^, 1646–1662 cm^−1^, 1662–1681 cm^−1^, and 1682–1700 cm^−1^ which belong to anti-parallel intermolecular β-sheet, intramolecular β-sheet, random coil, α-helix, β-turn, and parallel intermolecular β-sheet structures, respectively [55]. The α-helix is a repetitive helical conformation maintained by hydrogen bonding between the amino acid NH^−^ and the carbonyl oxygen C=O on the polypeptide chain. On the other hand, β-sheet structures are formed by multiple polypeptide chains (intermolecular β-sheet) or different peptides (intramolecular β-sheet) through hydrogen bonding, while β-turns are rotational structures connecting β-sheets using four amino acids under hydrogen bonding. The relative changes of different contents are related to the extension of the helical structure of the protein. It is generally recognized that α-helix and β-sheet belong to the relatively ordered secondary structure. In contrast, the β-turn and random coil are relatively disordered secondary structures.

**Figure 9:**
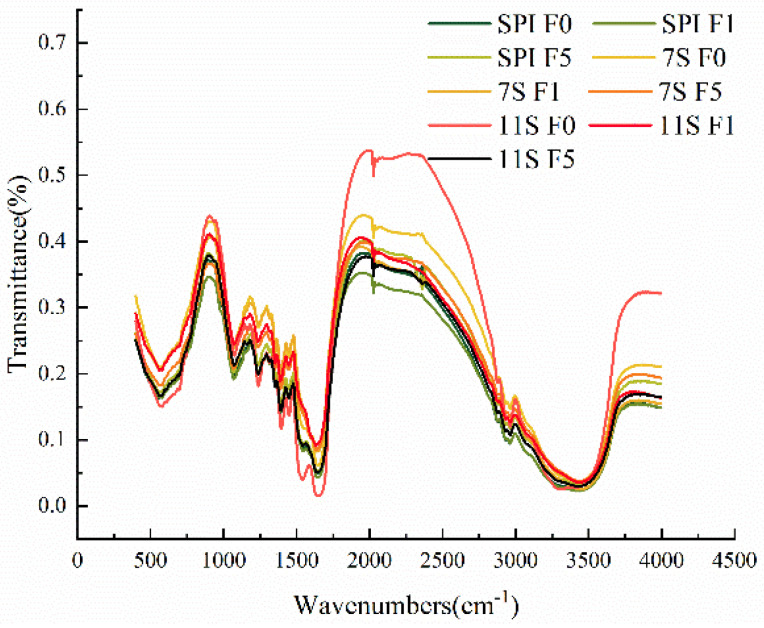
Fourier infrared spectra of protein solution frozen for different days.

According to Table 1 and Figure 9, the main protein structures of β-sheet and β-turn can be observed, with a low content of random coil. As the freezing time increased, the intensity of the hydroxyl group absorption peaks intensity of SPI decreased first and then increased, while it increased and then decreased for 7S and 11S. And the intensity of hydroxyl group absorption peak at 5 days of freezing was lower than that of unfrozen.

When unfrozen, the content of disordered structures of SPI, 7S, and 11S was 43.73%, 44.80%, and 45.08%, respectively, indicating that the structure of SPI was relatively more orderly and the protein structure was more stable. The β-sheet content of SPI was 30.81%, 30.15%, and 30.18%, respectively. The decline in β-sheet content indicated that the hydrophobic-associated sites of the protein were exposed, the hydrophobicity was enhanced, and the content of the ordered structure showed a decreasing trend. This change suggests that as the freezing time increases, the structure of SPI changed from ordered state to disordered state. This may be attributed to the crystallization of water during freezing, disrupting the association state of protein and water, and causing a decrease in the binding ability of protein and water and causing changes in the molecular structure of some proteins [56]. The content of α-helix and β-turn in 7S and 11S initially decreased and then increased, but the content after 5 days of freezing was still lower than that before frozen storage. The ordered structure content of 7S and 11S showed an increasing trend, indicating that during the freezing process, the hydrogen bonds that maintain the stability of α-helix structure were destroyed, the protein structure became loose, and the flexibility was enhanced. Esselink et al. [57] suggest that the rearrangement of protein spatial conformation during cryopreservation is caused by interaction between covalent and non-covalent bonds, such as hydrophobic interaction. The results indicate that ice crystals produced by cryopreservation cause the hydrogen bonds between amino acid residues inside the protein to be disrupted, the hydrophobic groups are exposed, and the protein molecules recombine, thus changing the secondary structure of the protein.

### 3.7. Total Sulfhydryl Content Analysis

**Figure 10:**
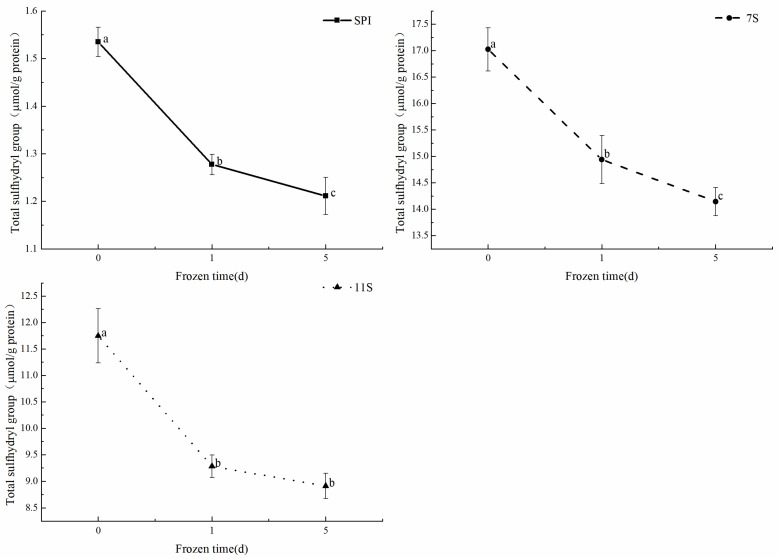
Changes in total sulfhydryl content of proteins under different days of freezing. Different lowercase letters on the shoulders of the same indicator indicate significant differences (*p* < 0.05).

The sulfhydryl group is known as one of the most reactive functional groups in proteins. As illustrated in Figure 10, when unfrozen, the total sulfhydryl content of SPI was only 1.53 μmol/g. The total sulfhydryl content of 7S was the highest at 17.02 μmol/g, while 11S had a total sulfhydryl content of 11.75 μmol/g. As the freezing time increased, the total sulfhydryl content of the proteins progressively decreased, following a similar trend to the alteration in soluble protein content. However, the decrease of total sulfhydryl content after 5 days of freezing was not as significant as the decrease observed after 1 day of freezing. Additionally, even after 5 days of freezing, the total sulfhydryl content of 7S was still higher than that of SPI and 11S without freezing, measuring 14.15 μmol/g. During freezing, the sulfhydryl content decreased by 21.10%, 16.91%, and 24.14%, respectively. These findings indicate that the freezing process breaks the hydrogen bonds of water molecules, disrupts the interaction between proteins and water in solution, changes the hydrophobic microenvironment of the original protein molecules, causing the exposure of some hydrophobic groups, which will cause rearrangement between proteins and lead to aggregation. The proximity of sulfhydryl groups caused by protein aggregation also increases the chance of disulfide bond formation, which leads to a decrease in sulfhydryl content [58]. It is also possible that protein oxidation during freezing may also lead to oxidation of some active sulfhydryl groups, resulting in the formation of disulfide bonds and a decrease in the total sulfhydryl content [59]. A similar trend was observed in the freeze–thaw cycle of cuttlefish flesh protein, where the total sulfhydryl content decreased continuously, indicating the transformation of buried sulfhydryl groups into disulfide bonds [60]. According to Liu, Qian et al., the total sulfhydryl and active sulfhydryl content of carp surimi gradually decreased with the increase of freezing time, and the protein denaturation and aggregation occurred [61], ultimately leading to a decrease in solubility.

### 3.8. Effect of Chemical Forces in Protein Solutions

**Figure 11:**
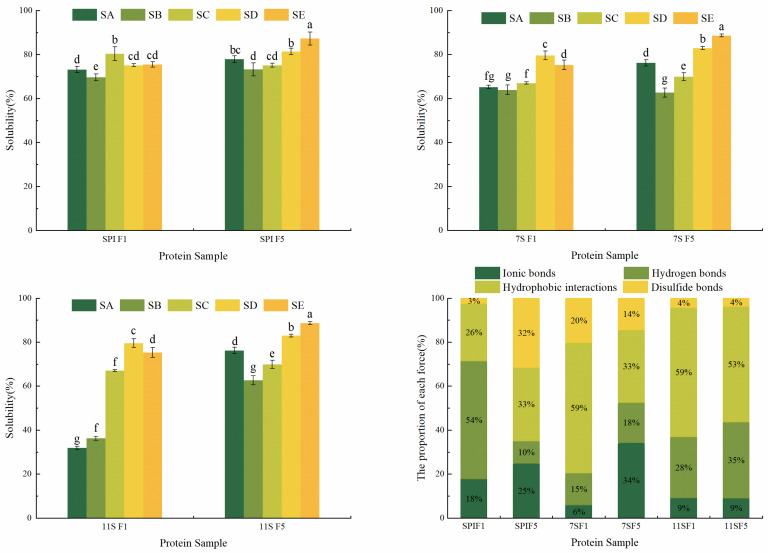
Effect of different days of freezing on the intermolecular forces of proteins. Different lowercase letters on the shoulders of the same indicator indicate significant differences (*p* < 0.05).

As shown in Figure 11, protein forces are primarily composed of ionic bonds, hydrogen bonds, hydrophobic interactions, and disulfide bonds, with hydrophobic interactions accounting for the largest proportion and being the most significant. The force of protein is calculated based on the difference of soluble protein content of protein in different solutions, so it can not reflect the absolute value of the force content of different proteins, but can only reflect the proportion of each force in different proteins. The solubility of SPI, 7S, and 11S in SA, SB, SC, SD, and SE solutions was the lowest in SB solution and the highest in SE solution. This suggests that hydrophobic interactions and disulfide bonds are particularly important in maintaining protein structure [62]. The contribution of hydrophobic interactions and disulfide bonds in SPI increased, leading to the exposure of hydrophobic groups on the protein surface, enhanced hydrophobicity, gradual exposure of tryptophan residues, and increased polarity of their microenvironment. Freezing promoted the oxidation of sulfhydryl groups in the protein, resulting in their conversion into disulfide bonds. This disruption of the tertiary structure [63] led to protein aggregation. The contribution of ionic bonds in SPI and 7S solutions increased. This may be attributed to the unfolding of protein molecular chains during freezing and the formation of new structures in the presence of salt ions. The hydrogen bonding contribution in 7S and 11S showed an increasing trend. Additionally, during freezing, the protein–protein and protein–water binding was enhanced due to the formation of ice crystals. As a result, the particle size of protein solution increased and the solubility decreased. These findings indicate that during freezing, different protein solutions are subjected to different forces to denature the internal structure of the protein.

## 4. Conclusions

Through a comparative analysis of the physicochemical properties of soybean isolate (SPI), ß-conglycinin (7S), and glycinin (11S) solutions before and after freezing, notable findings emerged. The solubility of the protein solutions demonstrated a decreasing trend with prolonged freezing time, with the most significant decline observed in 11S, followed by SPI, and the least in 7S. Freezing resulted in an increase in viscosity for all three protein solutions, exhibiting an inverse relationship with shear rate. Additionally, post-freezing, varying degrees of protein aggregation were observed, accompanied by an expansion of particle size distribution and an increase in average particle size, with the most pronounced effect seen in 11S, followed by SPI, and the least in 7S. During the freezing process, changes occurred in the internal microenvironment of the protein solution as water froze, leading to the exposure of hydrophobic groups and a red shift in the maximum absorption wavelength of the proteins. Moreover, as freezing time increased, the secondary structure of the protein transformed from a disordered state to an ordered state. This structural transition was accompanied by a decrease in the total sulfhydryl content of the protein, indicating the formation of new disulfide bonds between protein molecules during freezing. Force analysis highlighted hydrophobic forces as the primary driving force behind the formation of insoluble protein aggregates during freezing. In summary, freezing induces alterations in the hydrophobic microenvironment of soy protein solutions, resulting in changes in the natural conformation of proteins. These changes, in turn, facilitate the aggregation of protein molecules through hydrophobic interactions, ultimately leading to the formation of insoluble aggregates. Notably, due to inherent structural and molecular disparities, the three proteins (SPI, 7S, and 11S) exhibit distinct physical and chemical properties during freezing, with 11S displaying the most significant impact, followed by SPI, and 7S being the least affected. This study delves into the investigation of the physical and chemical property changes of these three proteins under freezing, providing a valuable theoretical foundation for enhancing the functional properties of soy protein and broadening its application in frozen food products.

## Figures and Tables

**Table 1:** Secondary structure distribution of protein solutions at different days of freezing.

	Seondary Structure					
	Anti-Parallel Intermolecular β-Sheet (%)	Intramolecular β-Sheet (%)	Random Coil (%)	α-Helix (%)	β-Turn (%)	Parallel Intermolecular β-Sheet (%)
SPI F0	4.82 ± 0.06 ^d^	19.48 ± 0.07 ^a^	12.27 ± 0.01 ^cd^	25.45 ± 0.03 ^bc^	31.46 ± 0.06 ^ab^	6.51 ± 0.07 ^b^
SPI F1	4.67 ± 0.05 ^e^	19.34 ± 0.06 ^a^	12.53 ± 0.02 ^abc^	25.98 ± 0.03 ^ab^	31.30 ± 0.08 ^ab^	6.14 ± 0.03 ^cd^
SPI F5	4.66 ± 0.03 ^e^	19.34 ± 0.04 ^a^	12.53 ± 0.00 ^abc^	25.97 ± 0.01 ^ab^	31.29 ± 0.04 ^ab^	6.18 ± 0.01 ^cd^
7S F0	3.88 ± 0.04 ^f^	18.62 ± 0.03 ^b^	12.76 ± 0.00 ^ab^	26.42 ± 0.03 ^a^	32.04 ± 0.06 ^a^	6.28 ± 0.02 ^bc^
7S F1	3.53 ± 0.21 ^g^	17.20 ± 1.02 ^c^	12.70 ± 0.72 ^abc^	24.82 ± 1.42 ^c^	29.50 ± 1.67 ^c^	6.39 ± 0.38 ^bc^
7S F5	4.93 ± 0.02 ^cd^	19.49 ± 0.02 ^a^	12.56 ± 0.02 ^abc^	25.54 ± 0.01 ^abc^	30.68 ± 0.02 ^b^	6.79 ± 0.01 ^a^
11S F0	11.50 ± 0.06 ^a^	11.46 ± 0.02 ^d^	12.98 ± 0.02 ^a^	26.00 ± 0.02 ^ab^	32.10 ± 0.06 ^a^	5.95 ± 0.03 ^d^
11S F1	6.07 ± 0.04 ^b^	19.43 ± 0.01 ^a^	11.87 ± 0.02 ^d^	24.87 ± 0.04 ^c^	30.75 ± 0.01 ^b^	7.01 ± 0.02 ^a^
11S F5	5.01 ± 0.05 ^c^	19.61 ± 0.01 ^a^	12.37 ± 0.03 ^bc^	25.78 ± 0.03 ^ab^	31.23 ± 0.04 ^ab^	5.98 ± 0.03 ^d^

Note: The results are expressed as M ± SD. M: average; SD: standard deviation. Note: different low-ercase letters on the shoulders of the same indicator indicate significant differences (*p* < 0.05).

## Data Availability

The data presented in this study are available on request from the corresponding author.
